# The Investigation of Hepatitis B Vaccine Immune Responses in Occult Hepatitis B Virus-Infected Patients

**DOI:** 10.3389/fimmu.2022.903685

**Published:** 2022-05-27

**Authors:** Jing Peng, Xueying Yao, Chunyan Yuan, Xiaoli Liu, Renxiang Xia, Jian He, Rui Li, Yunqing Yao

**Affiliations:** ^1^ The Department of Infectious Diseases, The First Affiliated Hospital of Chongqing Medical University, Chongqing, China; ^2^ The Department of Radiology, The Second Affiliated Hospital of Chongqing Medical University, Chongqing, China

**Keywords:** OBI, T lymphocyte, B lymphocyte, hepatitis B vaccine, anti-HBs, immunotherapy, therapeutic potential

## Abstract

**Objectives:**

There is no effective treatment for occult hepatitis B virus infection (OBI) patients, and immunotherapy may be one of the most promising options. We aim to investigate the underlying mechanism and therapeutic potential of hepatitis B vaccine immunotherapy for OBI patients.

**Methods:**

Outpatient OBI patients were screened and randomly divided into treatment (Group A) and control (Group B) groups. At weeks 0, 4, and 24, patients in Group A received a subcutaneous/intramuscular injection of hepatitis B vaccine (Engerix-B, 20 μg/time) according to the standard vaccination schedule; patients in Group B served as blank control. The patients were followed for 36 weeks, with clinical, biochemical, virological, immunological, and imaging data collected and analyzed at weeks 0, 12, 24, and 36, respectively, and the relation between the virology and immunology results was analyzed.

**Results:**

Of the 228 OBI patients, 28 were excluded, and 200 were enrolled for observation. In the end, 44 patients were included in Group A and 39 in Group B after excluding lost cases. At week 0 (baseline), some patients in two groups had liver disease symptoms, HBV-related liver function damage, and liver fibrosis. 86.36% (38/44) and 82.05% (32/39) patients were positive for serum hepatitis B surface antibodies (anti-HBs) in Group A and Group B, respectively, with the median (quartile) of 42.47 (16.85, 109.1) and 39.27 (16.06, 117.4) mIU/ml, respectively. Reduced peripheral blood CD4^+^T, CD8^+^T, and B lymphocytes were found in some patients in two groups. These results were not statistically different between Group A and Group B (*P>*0.05). At week 36, all patients were serum anti-HBs (+) in Group A, with a median (quartile) of 1000 (483.9, 1000) mIU/ml, which was significantly higher than that at week 0 (*P*<0.05) and that in Group B (*P*<0.05). Compared to week 0, the number of CD8^+^ T and B lymphocytes increased significantly and were significantly higher than Group B at the same point. Two patients in Group B were found to have hepatitis B virus reactivation from week 12 to week 36.

**Correlation Analysis:**

Anti-HBs in Group A patients were positively correlated with B lymphocytes (r=0.3431, 0.3087, and 0.3041, respectively) and positively correlated with CD8^+^ T lymphocytes (r=0.4954, 0.3054, and 0.3455, respectively) at weeks 12, 24, and 36.

**Conclusion:**

Virological reactivation is a risk for OBI patients. Serum hepatitis B surface antibodies were significantly increased after hepatitis B vaccine treatment, the same as the numbers of peripheral blood B and CD8^+^ T lymphocytes; changes in hepatitis B surface antibody levels were positively correlated with the changes in peripheral blood B and CD8^+^ T lymphocytes.

## 1 Introduction

There is about 296 million hepatitis B surface antigen (HBsAg) positive chronic hepatitis B virus infection (CHB) patients around the world, and approximately 820,000 people die each year from HBV-related liver cirrhosis or/and HBV-related hepatocellular carcinoma (HCC) (1). OBI is defined as serum HBsAg negative, hepatitis B core antibody (anti-HBc) positive, normal ALT values, and usually, but not always, undetectable serum HBV DNA, but HBV covalently closed circular DNA (cccDNA) was detectable in the liver, according to the latest guidelines from the European Association for the Study of the Liver (EASL) (2). HBV pregenomic RNA (pgRNA) and hepatitis B core-related antigen (HBcrAg) are reliable substitutes for cccDNA (2–6). The prevalence of OBI patients with HBsAg negative is not significant and varies by region and disease. The overall prevalence of OBI in Sudan was 15.51%, with a high level of heterogeneity (7). A meta-analysis showed that the overall prevalence of OBI in Western Europe and Northern America was 34%, 28% in 329 subjects without chronic liver disease, and 35% in 2400 patients with chronic liver disease (8). The prevalence of OBI is significantly different among patients with different diseases. That is, in patients with cryptogenic cirrhosis or advanced liver fibrosis, the prevalence of OBI ranges from 4% to 38%, in the case of parenteral blood exposure, it is about 45%, in patients with chronic hepatitis C, it is estimated at 52%, in HIV-infected patients, it ranges from 0% to 45%, in blood donors from 0% to 22.7% and in hemodialysis patients, it ranges from 0% to 54% (9). In local areas of China, the prevalence of OBI was 19.48% (640/3100 cases) in the young people without the hepatitis B vaccine and 4.70% (170/3615 cases) in that vaccinated with the hepatitis B vaccine (10). Our research team estimates at least 20 million OBI patients in China (11).

Currently, major international guidelines (2, 4, 5) believe that no treatment is necessary once CHB has achieved a “functional cure” (i.e., HBsAg-negative OBI status). However, even when HBsAg is negatively converted, cccDNA remains in the liver, and HBV replicates at a low level and integrates into the host (12), resulting in “asymptomatic” chronic inflammation. OBI is a unique type of HBV infection that is currently circulating. Our research team and others have discovered that OBI can cause HBV reactivation, liver cirrhosis, and HCC (11, 13, 14).

Chronic exposure to high levels of HBsAg causes immune cell depletion and inadequate responses in CHB. OBI had a significantly better immune response than CHB, but it was still below that of healthy people (15, 16). Restoring the immune function of HBV-infected patients might eradicate the virus (6). Anti-HBs are known to be protective antibodies (17), capable of preventing reinfection and maintaining host immunosuppression against HBV (18), and our research team discovered that anti-HBs might also prevent HCC progression (11). It is proposed to treat OBI with a recombinant yeast-derived hepatitis B vaccine, observe immunological changes, and investigate its therapeutic potential.

## 2 Materials and Methods

### 2.1 Study Objects

From November 2019 to November 2021, 228 OBI patients aged 18 to 65 admitted to the outpatient department of the Department of Infectious Diseases of the First Affiliated Hospital of Chongqing Medical University were screened, 28 cases were excluded, and 200 cases were enrolled for observation and randomly divided into treatment group (Group A) and control group (Group B). The study was conducted following the Declaration of Helsinki, and all participants signed informed consent forms.

#### (1) Criteria for Inclusion:

According to the EASL guidelines (2), the following criteria must be met: serum HBsAg negative (<0.05 IU/ml), HBeAg negative (<1S/CO), anti-HBc positive (>1S/CO) for more than 6 months, with/without anti-HBe positive (<1S/CO), with/without anti-HBs positive (≥10 mIU/ml); with/without serum HBV DNA positive (≥10 IU/ml); HBcrAg positive (≥3 LogU/mL); With or without imaging abnormalities.

#### (2) Criteria for Exclusion:

Co-infection with hepatitis A, B, C, D, E, HIV, Epstein-Barr virus, autoimmune hepatitis, hereditary liver disease, steatohepatitis, liver cirrhosis, HCC, and serious other system diseases, patients who received anti-HBV therapy or immunomodulatory treatment in 6 months, pregnant women, and lactating women.

### 2.2 Treatment Methods and Observation Indicators

#### (1) Treatment Methods:

The patients in Group A received a 20 μg/time injection of recombinant Saccharomyces cerevisiae-derived hepatitis B vaccine (Engerix B) subcutaneously/intramuscularly at weeks 0, 4, and 24, according to standard vaccination procedures. Patients in Group B served as blank control.

#### (2) Observation Indicators:

At weeks 0, 12, 24, and 36, the patients were observed, and the following data were collected: 1) Epidemiological and clinical information, such as a family history of hepatitis B, clinical symptoms, and signs. 2) Biochemistry, including blood tests, liver function (ALT, GGT, TBIL, Alb), renal function, and AFP. 3) Virological indicators: serum HBV DNA, HBcrAg, HBsAg, HBeAg, anti-HBs, anti-HBe, and anti-HBc; 4) Immunological indicators: total lymphocytes (tLymph), CD3^+^T, CD4^+^T, CD8^+^T, and B lymphocytes in peripheral blood; 5) Imaging data: color Doppler ultrasound of the upper abdomen/CT enhanced scan/MRI enhanced scan, liver transient elastography, and liver fat content detection. (Anti-HBs were expressed and calculated as 1000 mIU/ml when ≥1000 mIU/ml).

### 2.3 Statistical Analysis

GraphPad Prism 8.0.1 was used to conduct the statistical analysis. If the two groups of data have a normal distribution, the t-test was used; otherwise, the Wilcoxon test was used; when comparing multiple groups, the RM one-way ANOVA analysis was used if the data had a normal distribution. Otherwise, the Friedman analysis was used. The correlation analysis was performed with the Spearman. *P*<0.05 denotes a statistically significant difference.

## 3 Results

In the end, 117 cases were excluded from the 200 OBI patients. 44 cases in Group A and 39 cases in Group B were included in the statistical analysis.

### 3.1 Baseline Information at Week 0

#### 3.1.1 Some Patients in the Two Groups Had Liver Disease Symptoms

There was no statistical difference between Group A and Group B (*P>*0.05), as shown in **Table 1**.

**Table 1:** Baseline characteristics of OBI patients at week 0.

Items	Group A (n=44)	Group B (n=39)
**Mean age (± SD)**	49.55 ± 9.43	46.54 ± 10.42
**Male n° (%)**	22 (50.00)	20 (51.28)
**History of drinking n° (%)**	17 (38.64)	16 (41.03)
**History of smoking n° (%)**	14 (31.82)	14 (35.90)
**Family history of hepatitis B n° (%)**	20 (45.45)	18 (46.15)
**Liver disease related symptoms n° (%)**	7 (15.91)	6 (15.38)

n°, number of cases.

### 3.2 Results of Biochemical and Imaging Tests

#### 3.2.1 Some Patients in Two Groups Had HBV-Related Liver Function Damage and Liver Fibrosis at Week 0

From week 0 to week 36, all patients’ test results for blood routine, renal function, and alpha fetal protein (AFP) were normal. There were no vaccine-related adverse events found in Group A. There was no liver cirrhosis and HCC in the two groups.

At week 0, after removing hemolysis, drug/alcoholism, steatohepatitis, and other liver damage factors, 4.55% (2/44) of patients in Group A and 7.7% (3/39) in Group B had HBV-related liver function damage (one or more of ALT/GGT/TBIL abnormal, as shown in **Table 2**), and all returned to normal after treatment with liver-protective drugs within 24 weeks. There was no statistical difference between Group A and Group B (*P>*0.05).

**Table 2:** Results of biochemical and imaging examination in OBI patients at week 0.

Items	Group A (n=44)	Group B (n=39)
**Biochemical**		
**Alb, M (Q1,Q3), g/L**	49.00 (47.00,50.00)	48.00 (45.00,50.00)
**ALT, M (Q1,Q3), U/L**	20.50 (13.00,28.00)	22.00 (15.00,33.00)
**GGT, M (Q1,Q3), U/L**	20.50 (15.00,33.00)	24.00 (16.00,41.00)
**TBIL, M (Q1,Q3), μmol/L**	12.05 (8.15,16.68)	11.00 (7.10,14.00)
**HBV related liver dysfunction n° (%)**	2 (4.55)	3 (7.70)
**Imaging**		
**Hepatic fibrosis n° (%)**	27(61.36)	25(64.10)

M, median; Q1, first quartile; Q3, third quartile; n°, number of cases.

At week 0, liver fibrosis was found in 61.36% (27/44) and 64.1% (25/39) of Group A and B patients, respectively. There was no statistical difference between Group A and Group B (*P>*0.05), as shown in **Table 2**.

### 3.3 Virological Findings

#### 3.3.1 Serum Anti-HBs of OBI Patients in Two Groups Were Very Low at Week 0, But They Significantly Increased After HBV Vaccine Treatment in Group A; HBV Reactivation Was Observed in 2 Patients in Group B From Week 12 to Week 36

At week 0, all patients in Group A and Group B had serum anti-HBc(+) and HBcrAg(+), 52.27% (23/44) of patients in Group A and 61.54% (24/39) in Group B had anti-HBe(+). 86.36% (38/44) of patients in Group A and 82.05% (32/39) in Group B were anti-HBs(+), the median (quartile) of anti-HBs was 42.47 (16.85, 109.1) and 39.27 (16.06, 117.4) mIU/ml in all patients of Group A and Group B, respectively. There was no statistical difference between Group A and Group B (*P>*0.05). Serum HBV DNA, HBsAg, and HBeAg were negative, as shown in **Table 3**.

**Table 3:** Results of serum markers of hepatitis B virus in OBI patients.

Group	Time	HBcrAg(+)	HBV DNA(+)	HBsAg(+)	HBeAg(+)	anti-HBe(+)	anti-HBc(+)
		n° (%)	n° (%)	n° (%)	n° (%)	n° (%)	n° (%)
**Group A (n=44)**	**0 w**	44 (100.00)	N	N	N	23 (52.27)	44 (100.00)
	**12 w**	–	N	N	N	22 (50.00)	44 (100.00)
	**24 w**	–	N	N	N	22 (50.00)	44 (100.00)
	**36 w**	–	N	N	N	22 (50.00)	44 (100.00)
**Group B (n=39)**	**0 w**	39 (100.00)	N	N	N	24 (61.54)	39 (100.00)
	**12 w**	–	N	1 (2.56)	N	24 (61.54)	39 (100.00)
	**24 w**	–	1 (2.56)	2 (5.13)	N	24 (61.54)	39 (100.00)
	**36 w**	–	N	2 (5.13)	N	24 (61.54)	39 (100.00)

N, none; n°, number of cases; -, without detection.

At weeks 12, 24, and 36, 2 cases in Group B and 0 cases in Group A were tested for HBV DNA and/or HBsAg re-positive. After the treatment of the HBV vaccine, all patients in Group A were anti-HBs(+), with a median (quartile) of 1000 (483.9, 1000) mIU/ml at week 36, which was significantly higher than that at week 0 (*P*<0.05), also significantly higher than that in Group B (*P*<0.05). However, the rate of anti-HBs(+) in Group B had no significant changes compared with week 0, and neither did the anti-HBs titer (*P>*0.05), as shown in **Table 4**.

**Table 4:** Results of serum hepatitis B virus surface antibody test in OBI patients.

Group	Time	anti-HBs (+)				anti-HBs, (Q1, Q3), mIU/ml
		n° (%)				
		≥10 and <100	≥100 and <500	≥500 and <1000	≥1000	
		mIU/ml	mIU/ml	mIU/ml	mIU/ml	
**Group A (n=44)**	**0 w**	27 (61.36)	10 (22.73)	1 (2.27)	N	42.47 (16.85,109.10)
	**12 w**	6 (13.64)	9 (20.45)	2 (4.55)	27 (61.36)	1000 (230.4,1000)*
	**24 w**	4 (9.09)	7 (15.91)	5 (11.36)	28 (63.64)	1000 (485.1,1000)*
	**36 w**	4 (9.09)	7 (15.91)	4 (9.09)	29 (65.91)	1000 (483.9,1000)*
**Group B (n=39)**	**0 w**	20 (51.28)	12 (30.77)	N	N	39.27 (16.06,117.40)
	**12 w**	19 (48.72)	13 (33.33)	N	N	39.04 (16.09,112.60)
	**24 w**	20 (51.28)	12 (30.77)	N	N	40.48 (18.97,117.20)
	**36 w**	21 (53.85)	11 (28.21)	N	N	38.78 (16.18,115.20)

N, none; n°, number of cases; M, median; Q1, first quartile; Q3, third quartile; *, the difference was statistically significant compared with week 0 (*P*<0.05).

### 3.4 Immunological Findings

#### 3.4.1 At Baseline, the Number of Immune Cells Decreased in OBI Patients But Increased After HBV Vaccine Treatment

At week 0, the numbers of tLymph, CD3^+^ T, CD4^+^ T, CD8^+^ T, and B lymphocytes in peripheral blood were found lower than the Lower Limit of Normal (LLN, 1752, 1185, 561, 464, and 180 cells/μl, respectively) in 43.18% (19/44), 47.73% (21/44), 43.18% (19/44), 52.27% (23/44), and 38.64% (17/44) of patients in Group A and 41.03% (16/39), 46.15% (18/39), 35.90% (14/39), 58.97% (23/39), and 33.33% (13/39) of patients in Group B. There was no statistical difference between Group A and Group B (*P>*0.05), as shown in **Table 5**.

**Table 5:** Results of peripheral blood immune cell test in OBI patients.

Group	Time	tLymph	CD3^+^ T	CD4^+^ T	CD8^+^ T	B
		$\left(\bar{\chi }\pm \text{SD}\right)$ /μl	$\left(\bar{\chi }\pm \text{SD}\right)$ /μl	$\left(\bar{\chi }\pm \text{SD}\right)$ /μl	$\left(\bar{\chi }\pm \text{SD}\right)$ /μl	$\left(\bar{\chi }\pm \text{SD}\right)$ /μl
**Group A (n=44)**	**0 w**	1801.00±445.30	1210.00±371.90	646.10±240.80	459.50±191.70	232.30±100.70
	**12 w**	1926.00±486.90^a^	1291.00±376.90	666.70±258.90	516.60±192.70^ac^	245.90±101.20
	**24 w**	1898.00±423.30^a^	1316.00±362.00^a^	661.20±240.40	494.90±188.80a	245.50±115.80
	**36 w**	1853.00±446.80^a^	1241.00±357.80	645.80±232.10	487.70±188.50^ac^	249.30±107.90^ac^
**Group B (n=39)**	**0 w**	1803.00±482.00	1211.00±373.00	634.60±231.60	445.40±201.20	222.50±106.40
	**12 w**	1802.00±481.20	1205.00±372.60^b^	631.20±228.90^b^	430.50±191.20	211.20±110.50
	**24 w**	1791.00±484.90	1208.00±340.90	630.60±203.30	417.00±191.40^b^	210.80±84.59
	**36 w**	1782.00±470.40	1187.00±340.30	619.20±231.10^b^	404.10±189.80^b^	206.80±82.79

a The difference was statistically significant compared with week 0 in Group A (*P*<0.05);

b The difference was statistically significant compared with week 0 in Group B (*P*<0.05);

c The difference was statistically significant compared with Group B (*P*<0.05).

Compared with week 0, the number of tLymph in Group A patients was significantly increased at week 12, 24, and 36 (*P*<0.05); CD3^+^ T lymphocyte significantly increased at week 24 (*P*<0.05); CD4^+^ T lymphocyte increased at week 12 and 24 but decreased at week 36, and the difference was not statistically significant (*P*>0.05). CD8^+^ T lymphocyte was significantly increased (*P*<0.05) at week 12, 24, and 36; B lymphocyte was significantly increased at week 36 (*P*<0.05). Conversely, the number of lymphocytes in Group B showed a downward trend as a whole, among which CD3^+^ T lymphocytes decreased significantly at week 12 (*P*<0.05); CD4^+^ T lymphocytes decreased significantly at week 12 and 36 (*P*<0.05); and CD8^+^ T lymphocytes decreased significantly at week 24 and 36 (*P*<0.05).

The numbers of CD8^+^ T and B lymphocytes in Group A were significantly higher than in Group B at week 36 (*P*<0.05), as shown in **Table 5**.

### 3.5 Results of Correlation Analysis of Anti-HBs and Key Immune Cells:

#### 3.5.1 The Change of Anti-HBs Was Positively Correlated With the Change of CD8^+^ T Lymphocyte and B Lymphocyte in Group A During Treatment

**Figure 1:**
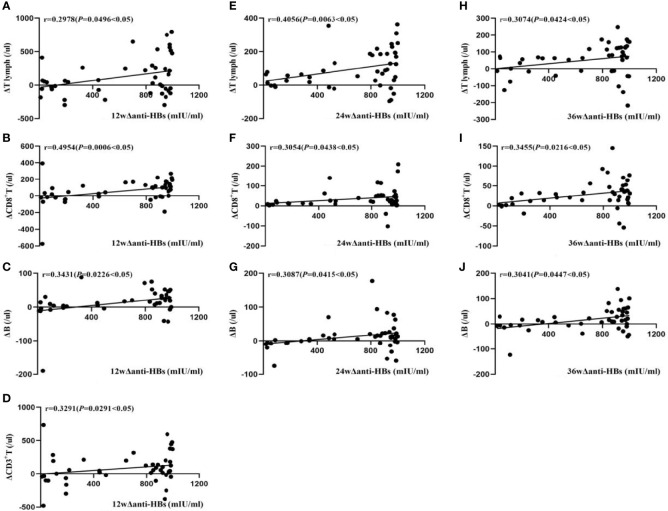
Correlation analysis of Δanti-HBs and Δimmune cells in Group A at week 12 **(A–D)**, week 24 **(E–G)**, week 36 **(H–J)**.

In Group A, at week 12, the changes of serum anti-HBs (Δanti-HBs) showed a moderate positive correlation with changes in CD8^+^ T levels (ΔCD8^+^T) (r = 0.4954, *P*<0.05), while they were weakly positively correlated with changes in peripheral blood tLymph levels (ΔtLymph), CD3^+^ T levels (ΔCD3^+^ T), and B lymphocyte levels (ΔB lymphocyte) (r = 0.2978, 0.3291, and 0.3431, respectively, *P*<0.05). The change in CD4^+^T level (ΔCD4^+^ T) did not correlate. At week 24, Δanti-HBs showed a moderate positive correlation with ΔtLymph (r = 0.4056, *P*<0.05), a weak positive correlation with ΔCD8^+^ T and ΔB lymphocyte (r = 0.3054 and 0.3087, *P*<0.05), but no correlation with ΔCD3^+^ T and ΔCD4^+^ T. At week 36, Δanti-HBs was weakly positively correlated with ΔtLymph, ΔCD8^+^ T, and ΔB lymphocyte (r=0.3074, 0.3455, and 0.3041, respectively, *P*<0.05)), but not with ΔCD3^+^T and ΔCD4^+^T cells, as shown in **Figure 1**.

**Figure 2:**
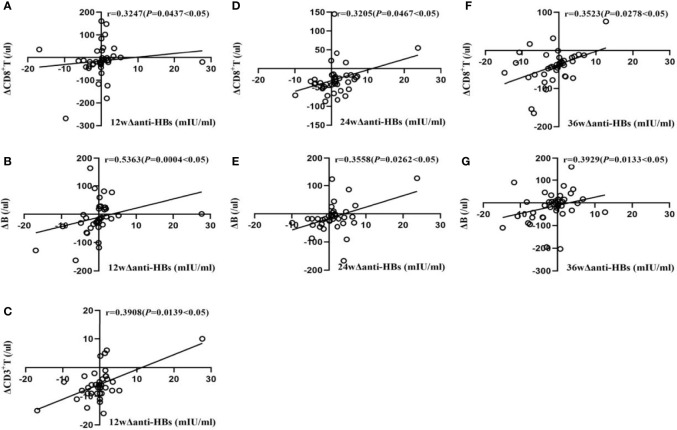
Correlation analysis of Δanti-HBs and Δimmune cells in Group B at week 12 **(A–C)**, week 24 **(D, E)**, week 36 **(F, G)**.

In Group B, at week 12, Δanti-HBs was moderately positively correlated with ΔB lymphocyte (r = 0.5363, *P*<0.05), weakly positively correlated with ΔCD3^+^ T and ΔCD8^+^T lymphocyte (r = 0.3908, and 0.3247, respectively, *P*<0.05); at week 24, it was weakly positively correlated with ΔCD8^+^ T and ΔB lymphocyte (r = 0.3205 and 0.3558, respectively, *P*<0.05); at week 36, it was weakly positively correlated with ΔCD8^+^ T and ΔB lymphocyte (r = 0.3523 and 0.3929, respectively, *P*<0.05), as shown in **Figure 2**.

## 4 Discussion

Patients with OBI are not safe, according to our findings. Nearly two-thirds of OBI patients have varying degrees of liver fibrosis. A small number of OBI patients have an abnormal liver function and liver disease-related symptoms, indicating that OBI is not in a “healthy state.” From 12 to 36 weeks of observation, serum hepatitis B surface antigen and HBV DNA reactivation were found in two patients of Group B, which meant that HBV in the OBI state is not always stable or cleared, consistent with our previous findings (11, 13).

According to our findings, the immune status of patients with OBI is subtle and complex. Before treatment, about half of patients with OBI had a lower number of CD8^+^ T lymphocytes in their peripheral blood than the lower limit of normal, about two-fifths of patients had a lower number of CD4^+^ T lymphocytes in their peripheral blood, and about a third of patients had a lower number of peripheral blood B lymphocyte, all of which are linked to the clearance process and pathogenic consequences of HBV. At the same time, even though more than three-quarters of OBI patients had positive serum anti-HBs, they all had low titers, which could be linked to a reduction in B cell number and/or function.

According to our findings, the hepatitis B vaccine can be used to treat and benefit patients with OBI. After hepatitis B vaccine treatment, the serum anti-HBs titer of OBI patients were significantly higher than week 0 and that of the control group, reaching the level of medium and high titers, significantly improving patient protection. At the same time, the serum hepatitis B surface antibody in the control group without hepatitis B vaccine treatment was always low positive rate and low level. Kato M et al. reported that serum high anti-HBs titers obtained after hepatitis B vaccination could prevent non-vaccine genotype HBV infection (19). Chen et al. also found that serum anti-HBs titers were inversely proportional to the risk of HBV reactivation following re-positivity of the surface antigen (20). In a previous study of OBI patients, our team discovered that the average diameter of HCC in anti-HBs-positive patients was significantly smaller than that in anti-HBs-negative patients (11).

The number of tLymph cells, CD8^+^ T lymphocytes, and B lymphocytes in the peripheral blood of OBI patients of the treatment group after 36 weeks of hepatitis B vaccine treatment were significantly increased and positively correlated with the levels of anti-HBs. We discovered that the hepatitis B vaccine not only activates B lymphocytes to produce anti-HBs but may also promote the activation and proliferation of CD8^+^ T lymphocytes directly or indirectly *via* anti-HBs, all of which aid the body’s eventual clearance of HBV. Contrarily, the number of peripheral blood immune cells in the control group was low during the observation period. We can only perform limited analysis with the results of other scholars’ studies in healthy and surface antigen-positive CHB patients because no similar studies have been reported. After hepatitis B vaccination, the number of B lymphocytes producing anti-HBs was positively correlated with the titer of anti-HBs (r=0.909) (21). After repeated hepatitis B vaccination, however, some CHB patients are unable to produce anti-HBs (22) because the number and function of HBsAg-specific B lymphocytes that produce anti-HBs are significantly reduced (21), and only a small amount of anti-HBs can be produced, resulting in the formation of immune complexes that are undetectable. When HBsAg is cleared, the total number of B lymphocytes in the peripheral blood increases (23), and HBsAg-specific memory B lymphocytes that have recovered their function can produce effective antibodies (24), or they can break the body’s immune tolerance to achieve clearance with the help of an effective therapeutic hepatitis B vaccine (25).

In conclusion, our ground-breaking research discovered that patients with OBI are not healthy, and their HBV is at risk of reactivation. The use of the hepatitis B vaccine in the treatment of OBI patients can increase serum hepatitis B surface antibody titers, reducing the risk of reinfection with other genotypes of HBV, as well as promote complete clearance of HBV by activating the body’s cellular and humoral immunity and even reduce the risk of HBV-related cirrhosis and HCC.

Our research still has room for improvement: many patients were lost to follow-up during the research process, and the number of cases available for statistical analysis needs to be increased. Treatment time and duration should be extended, and immunological mechanism research, liver histological examination, intrahepatic HBV-related virology, and immunological research should all be improved.

## Data Availability Statement

The raw data supporting the conclusions of this article will be made available by the authors, without undue reservation.

## Ethics Statement

The studies involving human participants were reviewed and approved by Ethics Committee of the First Affiliated Hospital of Chongqing Medical University. The patients/participants provided their written informed consent to participate in this study.

## Author Contributions

YY designed the project, revised the manuscript, and approved the submission; JP, XY, CY, and XL collected and analyzed case data and wrote the manuscript; RX, JH, and RL participated in collecting case data. All authors contributed to the article and approved the submitted version.

## Conflict of Interest

The authors declare that the research was conducted in the absence of any commercial or financial relationships that could be construed as a potential conflict of interest.

## Publisher’s Note

All claims expressed in this article are solely those of the authors and do not necessarily represent those of their affiliated organizations, or those of the publisher, the editors and the reviewers. Any product that may be evaluated in this article, or claim that may be made by its manufacturer, is not guaranteed or endorsed by the publisher.
